# A Universal Method to Weld Individual One-Dimensional Nanostructures with a Tungsten Needle Based on Synergy of the Electron Beam and Electrical Current

**DOI:** 10.3390/nano10030469

**Published:** 2020-03-05

**Authors:** Peng Zhao, Yu Zhang, Shuai Tang, Runze Zhan, Juncong She, Jun Chen, Shaozhi Deng

**Affiliations:** State Key Laboratory of Optoelectronic Materials and Technologies, Guangdong Province Key Laboratory of Display Material and Technology, and School of Electronics and Information Technology, Sun Yat-sen University, Guangzhou 510275, China; zhaop36@mail2.sysu.edu.cn (P.Z.);

**Keywords:** one-dimensional nanostructures, nanostructure welding technology, in-situ measurement, electron-beam-induced deposition (EBID), local current heating

## Abstract

One-dimensional (1D) nanostructures are extensively used in the design of novel electronic devices, sensors, and energy devices. One of the major challenges faced by the electronics industry is the problem of contact between the 1D nanostructure and electrode, which can limit or even jeopardize device operations. Herein, a universal method that can realize good Ohmic and mechanical contact between an individual 1D nanostructure and a tungsten needle at sub-micron or micron scale is investigated and presented in a scanning electron microscope (SEM) chamber with the synergy of an electron beam and electrical current flowing through the welded joint. The linear I‒V curves of five types of individual 1D nanostructures, characterized by in-situ electrical measurements, demonstrate that most of them demonstrate good Ohmic contact with the tungsten needle, and the results of in-situ tensile measurements demonstrate that the welded joints possess excellent mechanical performance. By simulation analysis using the finite element method, it is proved that the local heating effect, which is mainly produced by the electrical current flowing through the welded joints during the welding process, is the key factor in achieving good Ohmic contact.

## 1. Introduction

In recent decades, one-dimensional (1D) nanostructures have become the subject of extensive research, owing to their unique and excellent electrical, optical, thermal, mechanical, and magnetic properties. To date, many literature studies have reported the potential applications of 1D nanostructures in new nanoelectronic devices [1,2,3], optoelectronic devices [4,5], solar cells [6,7,8], energy storage devices [9,10], biosensors [11,12], chemical sensors [13,14], and field emission emitters [15,16]. To utilize the excellent properties of 1D nanostructures in the electronic devices, nanostructure welding technology, which can realize good Ohmic and machinal contact between an 1D nanostructure and an electrode, has been one of the most important processes in the application of 1D nanostructures. To date, there exist several methods that can be used to achieve good Ohmic contacts. For example, by rapid thermal annealing at 600–800 °C for 30 s, the contact resistances of carbon nanotube (CNT) devices have been reduced [17]. Dong et al. developed a method to reduce contact resistance between CNTs and metal electrodes by local Joule heating [18]. Suyatin et al. reported a method for the formation of Ohmic contacts to InAs nanowires by chemical etching and passivation of the contact areas in a mixture of ammonium polysulfide, (NH_4_)_2_S_x_ and water [19]. The methods reported above usually require tedious procedures, such as electron-beam (e-beam) lithography, metal deposition, and removal of e-beam resist, which risk contamination of the nanowire surfaces by the photoresist.

With the rapid development of technology, a more convenient method has been reported, using focused-ion-beam (FIB)-induced metal deposition on top of the nanostructures. For instance, Dong et al. demonstrated direct FIB nanopatterning of low-resistance Ohmic Pt contacts on n-type GaN nanowires [20]. It is well known that this method needs to be equipped with a costly FIB system in a high-resolution scanning electron microscope (SEM). In addition, several groups have also reported a series of studies to achieve nanostructure welding under in-situ conditions. For example, Chen et al. developed a method using an e-beam by exposing the contact area of the tip to establish Ohmic contacts on a CNT [21]. Zhang et al. demonstrated an electron point source by using the electron-beam-induced carbon deposition to fix a lanthanum hexaboride (LaB_6_) nanowire onto a tungsten needle [16]. Most investigations so far have been focused on the problem of Ohmic contact between the 1D metallic nanostructures and metal electrode, and methods for achieving good Ohmic contact and mechanical contact between 1D semiconductor nanostructures and metal electrodes under in-situ conditions have not been reported. Furthermore, there is a lack of further analysis on the factors influencing the nanostructure welding processes.

In this paper, a nanostructure welding method which can establish reliable Ohmic contact and mechanical contact using e-beam-induced deposition and the local heating effect is presented. With the welding process, various individual 1D nanostructures of different orientations can be conveniently realized on a tungsten needle. Meanwhile, the in-situ electrical and mechanical characterizations of five types of individual 1D nanostructures, including LaB_6_ nanowire, CNT, zinc oxide (ZnO) nanoneedle, silicon carbide (SiC) nanowire, and copper oxide (CuO) nanowire, are demonstrated on a nanomanipulation system operating inside an SEM. The results show that the nanostructure welding method is able to establish good Ohmic contact between the five types of 1D nanostructures and tungsten needles, which exhibit excellent mechanical performance as well. Furthermore, to explore the respective roles of the electron beam and electrical current flowing through the welded joints during the welding process, a numerical model based on the finite element method is built. The simulation results show that the local heating effect plays an important role in the nanostructure welding process. The findings can provide a universal method for welding individual 1D nanostructures onto metal electrodes with good Ohmic contact and excellent mechanical performance.

## 2. Experimental Section

### 2.1. Nanostructure Welding Processes, Morphology Characterizations, and Element Analysis of Individual 1D Nanostructure

The nanostructure welding was carried out in an SEM vacuum chamber with an in-situ characterization instrument comprising four mechanical manipulators under a vacuum level of 1 × 10^−3^ Pa. The experimental procedures of nanostructure welding included five steps. First, the tungsten needle was electrochemically sharpened to about 100 nm in diameter in NaOH solution (2 mol/L). Second, to improve the electrical conductivity of the tip, the W tip was melted into a ball with a clean and smooth surface by passing a large current through it. Third, an appropriate 1D nanostructure growing on the substrate was picked up by the melted tungsten needle, where the desired angle between the 1D nanostructure and the tungsten needle was controlled by the mechanical manipulator. Fourth, an e-beam, accelerated by voltages varying from 10–20 kV, was focused on the contact area of the 1D nanostructure and the tungsten tip, and an appropriate voltage was applied between the tungsten needle and the substrate using a Keithley 6487 Picoammeter. Simultaneously, the picoammeter read the current from the substrate, which was equal to the electrical current flowing through the welded joints. In this step, the electrical current flowing through the welded joints should be controlled appropriately for establishing good Ohmic contact, which was higher than 5 μA in our experiment. Furthermore, the deposition time of amorphous carbon induced by the e-beam should be appropriate for building good mechanical contact, which was more than 2 h in our experiment. Fifth, when good Ohmic and mechanical contacts were formed at the contact area, the individual 1D nanostructure was cut off by another two tungsten needles in the appropriate position by Joule melting. After the nanostructure welding process, the morphology of the individual 1D nanostructure was characterized using an SEM (Zeiss Supra 55, Carl Zeiss AG, Oberkochen, Germany). The structure information and element distribution of the nanostructure welding point were also analyzed using a transmission electron microscope (TEM, FEI Titan3 G2 60-300, FEI, Hillsboro, OR, USA) operated at 300 kV.

### 2.2. In-Situ Electrical Measurement of Individual 1D Nanostructure in an SEM

In-situ electrical measurements were carried out in an SEM vacuum chamber at a pressure of 5 × 10^−4^ Pa. Specifically, using two mechanical manipulators with a minimum moving step of 10 nm, an individual 1D nanostructure was placed and soldered between two different tungsten needles using the e-beam-induced carbon deposition [21]. A proper voltage, ranging from negative to positive voltages supplied by a Keithley 6487 Picoammeter, was applied to the 1D nanostructure through the two tungsten needles to obtain I‒V curves.

### 2.3. Simulation Methods

The temperature distribution of the individual 1D nanostructure under the synergy of the electron beam and electrical current flowing through the welded joints was calculated using the Comsol Multiphysics package. The program utilizes Finite Element Method. A physical model of heat transfer in solids was used to model the heat transfer in the whole welded structure mediated by conduction, convection, and radiation. It is worth mentioning that to compute the heat sources brought by electron beams, the node models of deposited beam power under the heat transfer model was used. In addition, a physical model of electrical transport, found under the AC/DC branch, was used to compute the Joule heat generated by the electrical current running through the whole welded structure. The simulation included three parts: First, the physical model was chosen, and geometric structures were built according to the corresponding actual test structures. Then, the electrical and thermal parameters of each material were entered into the model. Subsequently, the boundary conditions were set and the heat flux boundary with a Gaussian distribution of circle type was used to simulate the heating effect of the electron beam. Finally, the geometric structures were meshed appropriately, and the results were calculated.

## 3. Results and Discussion

### 3.1. Welding Process of Individual 1D Nanostructures with Different Orientations

For 1D nanostructures, their growth directions generally vary and are difficult to control. In different test scenes for in-situ measurement, the control of the angle between an individual 1D nanostructure and the tungsten needle should be considered. To solve this problem, according to our prior experiences, the nanostructure welding process of an individual 1D nanostructure was realized in two orientations—the nanowire was either perpendicular or parallel to the tungsten needle. Detailed nanostructure welding steps for one of the two orientations are demonstrated below.

**Figure 1:**
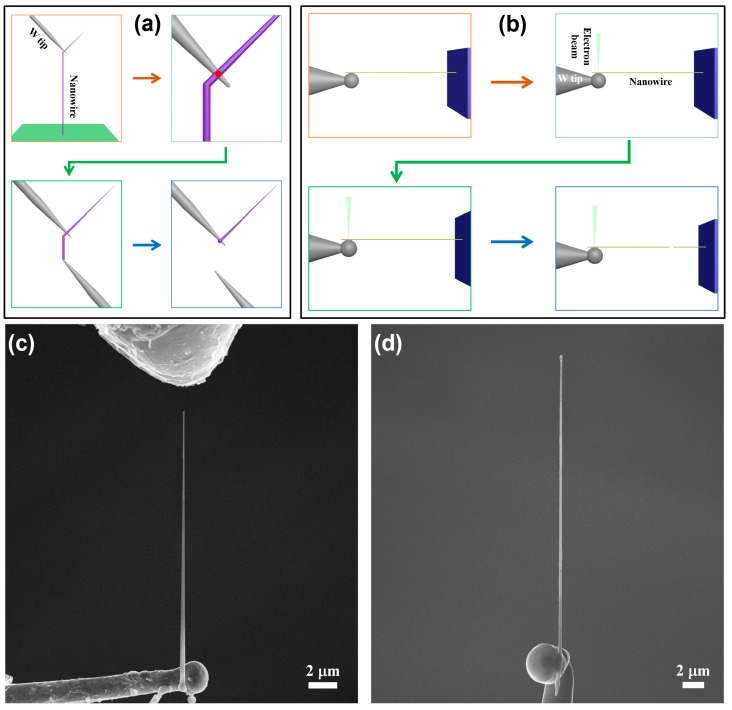
(**a**) Schematic diagram of the detailed nanostructure welding process in perpendicular orientation (red-spot area is the position of electron-beam irradiation). (**b**) Schematic diagram of the detailed nanostructure welding process in parallel orientation. (**c**) SEM image of an individual LaB_6_ nanowire welded on a tungsten needle in perpendicular orientation. (**d**) SEM image of an individual LaB_6_ nanowire welded on a tungsten needle in parallel orientation.

The welding process of an individual 1D nanostructure with different orientations is shown in Figure 1. The differences between the two procedures occurred mainly in the pick-up and cut-off steps. For example, in the pick-up step, the 1D nanostructure perpendicular to the tungsten needle was selected to obtain the perpendicular orientation, while the 1D nanostructure parallel to the tungsten needle was selected to obtain the parallel orientation. For the sake of simplicity, we took the welding procedure in perpendicular orientation as an example to describe the steps in detail, which is shown in Figure 1a.

The experimental procedure for nanostructure welding with perpendicular orientation included four steps. First, the tungsten needle controlled by the mechanical manipulator was moved to touch the appropriate position of an individual 1D nanostructure growing on a substrate. In this step, a van der Waals contact between the tungsten needle and individual 1D nanostructure was formed, and the contact performance was poor. Second, a voltage was applied between the tungsten needle and the individual 1D nanostructure. The current passing through the nanowire was measured by the Picoammeter. Meanwhile, an e-beam focused on the spot irradiated the contact area to generate an electrical current flowing through the welded joints. In this step, the welding process of the 1D nanostructure presented the following phenomena: (i) at the beginning of the electron-beam irradiation, the current was lower than 0.1 μA for voltages between 3 and 5 V; (ii) after irradiation by the electron beam for a short period (within a minute), the current went rapidly up to 1–100 μA. These phenomena indicated that the radiolysis effects of e-beam play a certain role in constructing a complete current circuit at the beginning of welding. To avoid a breakdown of the nanostructure, the voltage should be reduced at this point, such that the current going through the nanowire is within a safe range (for LaB_6_ and CNT, the current was in the range of 10–50 μA; for ZnO and SiC nanowire, the current was kept within 5 μA); and (iii) after electron-beam irradiation for several hours, an amorphous carbon layer produced by the decomposition of hydrocarbon molecules under the e-beam irradiation was deposited on the contact area. Such a process was to enhance the mechanical performance of the contact position. Third, after forming Ohmic contact between the nanowire and the tungsten needle, the nanowire was separated from the substrate by pulling it backward using a mechanical manipulator. Another tungsten needle was then employed to touch the broken position of the nanowire. The second step was repeated to ensure that good contact was formed between the nanowire and the tungsten needle. Fourth, a large voltage was applied to the two tungsten needles, such that the nanowire between the needles was fused by the large current passing through them. Finally, an individual 1D nanostructure perpendicular to the tungsten needle was obtained.

Figure 1b shows the detailed steps of nanostructure welding in the parallel orientation. It is similar to the above welding procedure, except that the nanowire is in parallel with tungsten needle in the first step. At the end of the welding procedure, the desired nanowire length can be obtained on the tungsten needle by using another two tungsten needles to form a loop current and fuse out the unwanted nanowires. Figure 1c,d show typical SEM images of individual LaB_6_ nanowires welded onto tungsten needles in different orientations.

### 3.2. Electrical Properties of the Individual 1D Nanostructures.

To acquire the electrical contact performances of the 1D nanostructures upon the above in-situ welding processes, the electrical properties of the individual 1D nanostructures were measured using a two-tungsten-needle probe system. Ohmic contacts with most of them were established and repeatable results were obtained.

#### 3.2.1. LaB_6_ Nanowire

**Figure 2:**
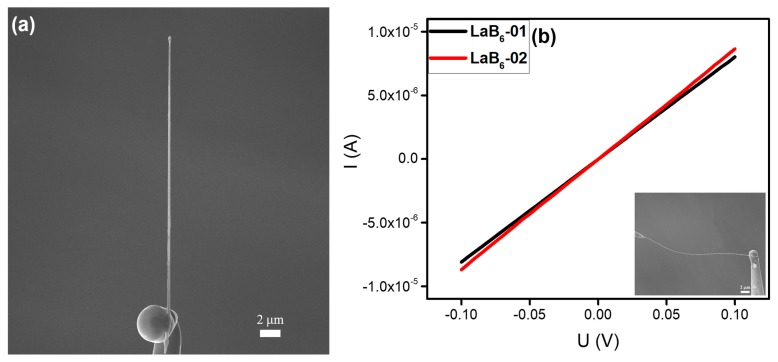
(**a**) SEM image of an individual LaB_6_ nanowire welded onto a tungsten needle. (**b**) Typical current versus voltage (I‒V curves) of two individual LaB_6_ nanowires. Typical SEM image of the in-situ electrical measurement is given as an inset.

Due to its high melting point, large electrical and thermal conductivities, low work function (~2.67 eV), and strong endurance in harsh environments [16,22,23,24], LaB_6_ has been widely applied for high electron density cathodes. Previously, LaB_6_ nanowires have been obtained by a simple one-step chemical vapor deposition (CVD) method [25]. As shown in Figure 2a, an individual LaB_6_ nanowire with a length of about 27 μm and a diameter of ~192 nm was placed and soldered on the top of a tungsten needle by the nanostructure welding process. Then, another tungsten needle was contacted and welded with the LaB_6_ nanowire for the in-situ electrical measurements. A voltage ranging from −0.1 to +0.1 V was applied to the LaB_6_ nanowire through the two tungsten needles to obtain I‒V curves, which are shown in Figure 2b. The linear I‒V curves obtained suggest an Ohmic contact between the tungsten needles and nanowire. The resistances calculated from these curves are about 12.3 and 11.5 kΩ, respectively, indicating that good Ohmic contacts had been built with the LaB_6_ nanowire. These results are repeatable.

#### 3.2.2. CNT

**Figure 3:**
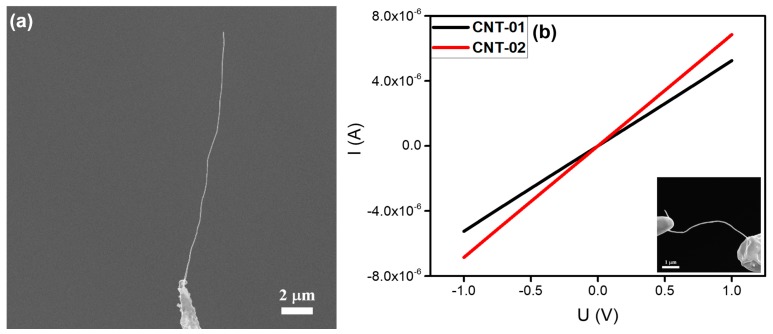
(**a**) SEM image of an individual carbon nanotube (CNT) welded onto a tungsten needle. (**b**) Typical current versus voltage (I‒V curves) of two individual CNTs. Typical SEM image of the in-situ electrical measurement is given as an inset.

CNT has attracted considerable attention due to its unique physical and electrical properties as well as its extensive potential applications [26,27,28]. In our experiments, the CNTs were prepared by thermal CVD using a 304 SS disc as the substrate [29]. As shown in Figure 3a, an individual CNT with a length of about 18 μm and a diameter of ~80 nm was welded on the top of a tungsten needle. The CNT was flexible and showed a curved shape. Similarly, in-situ electrical measurements were carried out to obtain I‒V curves, as shown in Figure 3b. A voltage ranging from -1 to +1 V was applied to the CNT and the I‒V curve was linear. The resistances calculated from these I‒V curves were about 190.3 and 145.8 kΩ, respectively, indicating that good Ohmic contacts had been made between the CNT and tungsten needle. These results were found to be repeatable.

#### 3.2.3. ZnO Nanoneedle

**Figure 4:**
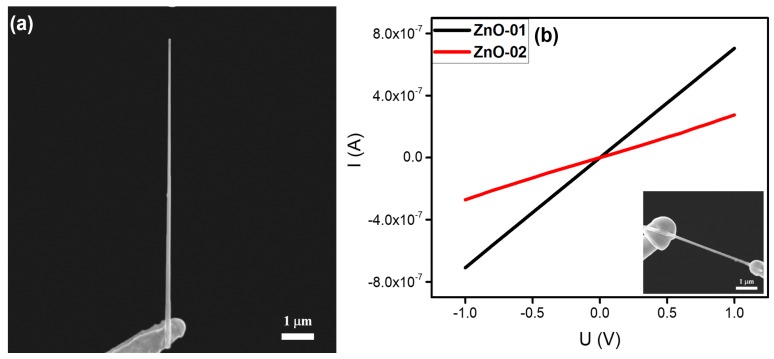
(**a**) SEM image of an individual ZnO nanoneedle welded onto a tungsten needle. (**b**) Typical current versus voltage (I‒V curves) of two individual ZnO nanoneedles. Typical SEM image of the in-situ electrical measurement is given as an inset.

At present, ZnO is one of the most important nanomaterials in nanotechnology research, owing to its relatively low production cost, direct wideband gap energy (3.37 eV), and superior optical properties [30,31,32]. In this work, ZnO nanoneedles were commercially obtained from the Jiangsu XFNANO Materials Tech Co. Ltd. As shown in Figure 4a, the sample had a tapered structure with a length of about 9 μm, a top diameter of ~70 nm, and a root diameter of ~160 nm. Furthermore, it presented good alignment relative to the substrate. The in-situ electrical measurement results on individual ZnO nanoneedles are shown in Figure 4b. The current increased linearly as the voltage was increased from −1 to +1 V. The calculated resistances were about 1.4 and 3.7 MΩ, respectively, indicating that good Ohmic contacts had been established between the ZnO and tungsten needles.

#### 3.2.4. SiC Nanowire

**Figure 5:**
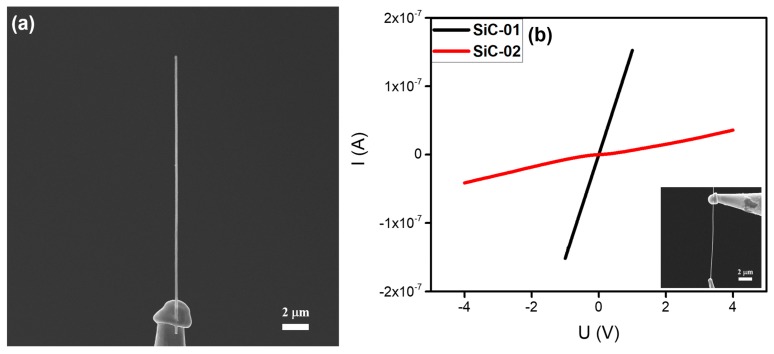
(**a**) SEM image of an individual SiC nanowire welded onto a tungsten needle. (**b**) Typical current versus voltage (I‒V curves) of two individual SiC nanowires. Typical SEM image of in-situ electrical measurement is given as an inset.

SiC is known as a significant wide-bandgap semiconductor material, owing to its high thermal conductivity, low thermal expansion coefficient, and good chemical stability [33,34,35]. In this work, SiC nanowires were commercially obtained from the Sinet Advanced Materials Co. Ltd. A typical individual SiC nanowire with a length of about 20 μm and a diameter of ~156 nm, which was welded onto the top of a tungsten needle, is shown in Figure 5a. The in-situ electrical properties of the individual SiC nanowire are shown in Figure 5b.

It is worth mentioning that the I‒V curves of the two SiC nanowires tested demonstrated different trends of resistance. As indicated in Figure 5b, the I‒V curve of sample SiC-01 was linear and the resistance at 1 V was calculated to be about 6.6 MΩ. This means that good Ohmic contact had been made between the SiC-01 and tungsten needle. However, the I‒V curve of the sample SiC-02 was non-linear at a low voltage range, indicating that a Schottky barrier had been formed between the SiC-02 and the tungsten needle. According to the linear region of the I‒V curve at high voltage, the resistance of the SiC-02 was calculated to be 86.3 MΩ. Thus, when the resistance of a SiC nanowire is larger than 10^7^ Ω, an Ohmic contact is difficult to form, and the welded joints between tungsten needle and SiC nanowire will be converted from Ohmic contact to Schottky contact. These results were repeatable.

#### 3.2.5. CuO Nanowire

**Figure 6:**
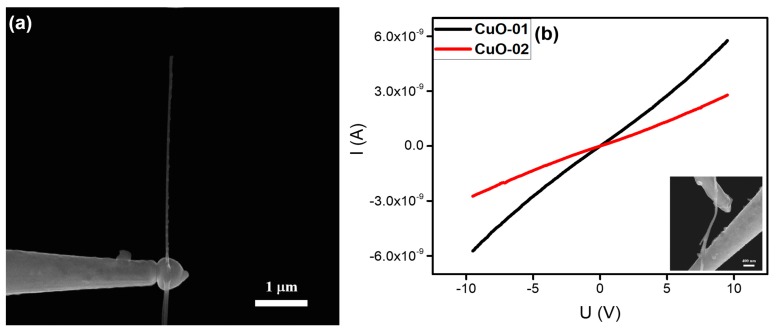
(**a**) SEM image of an individual CuO nanowire welded onto a tungsten needle. (**b**) Typical current versus voltage (I‒V curves) of two individual CuO nanowires. Typical SEM image of the in-situ electrical measurement is given as an inset.

CuO is known as a good candidate for photovoltaic devices, catalysts, sensors, stable electron sources, and optoelectronic devices owing to its direct narrow band gap, ultrahigh electrocatalytic activity, and stability [36,37,38]. In this work, CuO nanowires were grown using the thermal oxidation method [39]. An individual CuO nanowire with a length of about 4.5 μm and a diameter of ~43 nm, which was welded on the top of a tungsten needle, is shown in Figure 6a, which indicates that the CuO nanowire may have a large resistance. The above idea was confirmed by the I‒V curve in Figure 6b. The resistances at a lower voltage range were calculated to be about 1.9 and 3.9 GΩ, respectively, indicating that Ohmic contacts were formed between the CuO nanowires and the tungsten needle.

### 3.3. Theoretical Analysis of the Nanostructure Welding Process of the Individual 1D Nanostructure

**Figure 7:**
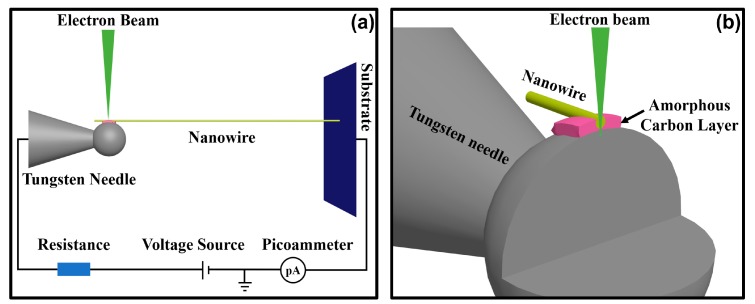
(**a**) Schematic diagram of the in-situ nanostructure welding process for an individual nanowire and its external circuit. (**b**) Schematic diagram of the structure inside the welded joints upon the nanostructure welding process.

The results of the in-situ electrical measurements demonstrate that the nanostructure welding process is a universal method to realize good Ohmic contact between individual 1D nanostructures and micro–nano tungsten needles. To understand the formation mechanism of the Ohmic contact, the effects of various factors in the nanostructure welding process were analyzed. As indicated in Figure 7a, the dominating factors in the nanostructure welding process were the electron beam and electrical current flowing through the welded joints. As mentioned in the literature [40], an electron beam can be focused onto diameters in the range of 0.8–2 nm, and about 95% of the electron kinetic energy is converted into heat. Thus, the resulting power density can be as high as 10^12^ W/m^2^. In addition, as shown in Figure 7b, as the penetration depth of the electron beam in the solid material can be larger than 1 μm, a current path was built at the contact point to create an electrical current flowing through the welded joints, provided by an external circuit. The initial contact point is poorly conductive, and the Joule heat generated by the electrical current is accumulated continuously at the contact point until the nanostructure welding process is completed.

#### 3.3.1. Effects of Local Joule Heating on Synergy of Electron Beam and Electrical Current

In our experiment, if the e-beam was irradiated onto the contact area without applying an external voltage, we found that it was much easier to build a Schottky contact than a good Ohmic one. Similarly, if the electron beam was not irradiated at the contact area initially, the electrical current flowing through the welded joints was very low (at 10 nA level) even at a voltage of 5 V. This situation did not change even after the voltage had been added to the tungsten needles for more than 10 min. Only upon irradiation of the e-beam onto the contact area did the electrical current induced by such an irradiation start to work. Thereafter the electrical current increased rapidly until reaching the maximum allowed by the current-limiting resistor. Therefore, it is noted that the irradiation of the e-beam was also indispensable, particularly at the beginning of the welding process, which is an important part for constructing the external current circuit. Only under the irradiation of the electron beam could the external circuit constructed form a complete current loop, and therefore achieve the formation of good Ohmic contact at the welded joint. As reported by the literature [41], structural and chemical changes were present in the oxide thin film induced by the radiolysis effects of the e-beam, resulting in the alteration of the electrical properties of amorphous oxide materials. We speculate that this behavior supports the explanation of those phenomena at the beginning of the welding process, which also exhibit strong beam sensitivity and the corresponding changes at the welded joint. After several hours, Ohmic contact was built by continuous electron-beam irradiation and the electrical current flowing.

**Figure 8:**
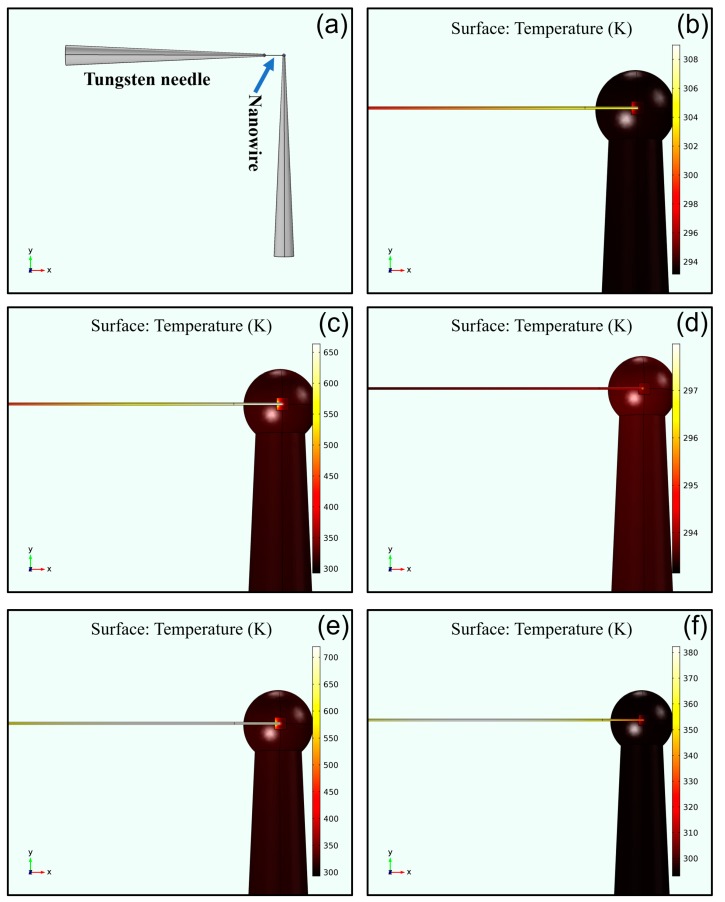
(**a**) Schematic diagram of the welded structure with an individual nanowire. (**b**–**d**) Temperature distributions of the LaB_6_ nanowire near the welded joint: (**b**) under the action of the e-beam radiation without an electrical current; (**c**) under the action of the e-beam radiation with an electrical current of 5 μA flowing through the welded joint; and (**d**) The Ohmic contact built after thermal equilibrium. (**e,f**) Temperature distributions of the SiC nanowire near the welded joint: (**e**) under the action of the e-beam radiation with an electrical current of 5 μA flowing through the welded joint; and (**f**) under the action of the e-beam radiation with an electrical current of 1 μA flowing through the welded joint.

To explore the local heating effects of the electron beam and the electrical current, the most direct way is to measure the temperature of the welded joint. However, temperature measurements on sub-micron areas are difficult under existing conditions. Therefore, using the finite element method to simulate the heating effect and temperature distribution of the welded joints was a viable solution. To compare the differences between metal-like nanowires and semiconductor nanowires in the nanostructure welding process, we selected the LaB_6_ nanowire and SiC nanowire as representatives for simulating the heating effect and temperature distribution. The results are shown in Figure 8. As mentioned in the literature, the thermal conductivities of the LaB_6_ and SiC were respectively 110 and 210 W/(m∙K) [42,43]. Moreover, the electrical conductivity of LaB_6_ nanowire was calculated as 4.3 × 10^5^ S/m by the I‒V curves in Figure 2, and that of the SiC nanowire was calculated as 3.0 × 10^2^ and 38 S/m by the I‒V curves in Figure 5. Besides these key factors, the intermediate layer of the amorphous carbon generated by the e-beam-induced deposition had a crucial impact on the results of the simulations. However, the electrical conductivity of the amorphous carbon was determined primarily by the number of sp^2^ sites existing in the material. As mentioned in the literature, the electrical conductivity of amorphous carbon is in the order of 10^−9^ S/m [44] and its thermal conductivity is in the order of 2.2 W/(m∙K) [45].

Figure 8a shows a schematic diagram of the welding structure to be simulated. The two cones are tungsten needles and the middle-elongated part is a nanowire. Figure 8b is the simulation results of the LaB_6_ nanowire near the welded joint under the action of e-beam radiation without an electrical current. As can be seen from Figure 8b that the highest temperature at the welded joint was about 309 K, which was much lower than the structure transformation temperature of the intermediate layer. Furthermore, the heating energy of the e-beam was assumed to be a disk with a Gaussian distribution on the welded joint in the simulation, whereas most of energy conversion actually occurred in the tungsten needle. This means that the temperature at the welded joint in the actual welding process was lower than the value of simulation. These results show that the local heating effect generated by the e-beam was not sufficient to establish a good Ohmic contact at the welding position, and the Joule heat generated by the electrical current flowing through the nanowires and the welded joint was the main heating source of the local heating effect. Figure 8c shows the simulation results of the LaB_6_ nanowire under the action of the e-beam radiation and an electrical current of 5 μA. The maximum temperature at the welded joint was more than 650 K, at which the composition of the intermediate layer changed such that the electric resistance of the intermediate layer was reduced and the heat conduction performance was improved. Then, the temperature at the welded joints decreased and good Ohmic contact was built after thermal equilibrium. Figure 8d shows the simulation result of the LaB_6_ nanowire near the welded joint with Ohmic contacts.

For the SiC nanowire, the resistance limited by its low electrical conductivity was much larger than that of the LaB_6_ nanowire. To obtain large enough electrical current flowing through the welded joints, the voltage of the external circuit had to be increased accordingly. However, the imaging effect of SEM is be affected when the voltage applied to the tungsten needle is too high. Therefore, to ensure that the e-beam was irradiated at the contact area by SEM imaging, the voltage of the external circuit had to be limited to 50 V. For the high-resistance SiC nanowire, the maximum current could only reach about 1 μA. Figure 8e shows the simulation results of the SiC nanowire near the welded joint under the action of the e-beam radiation with a 5 μA electrical current. In this situation, the resistance of the SiC nanowire was low and the maximum temperature at the welded joint was more than 700 K. On the contrary, as shown in Figure 8f, the maximum temperature at the welded joint with the high-resistance SiC nanowire was lower than 380 K. It is noteworthy that the highest temperature of the whole welded structure no longer appeared at the welded joint but, instead, appeared in the middle area of the SiC nanowire. Combined with the previous results shown in Figure 5b, it can be seen that the welded joints preferred to form Ohmic contacts, rather than Schottky contacts, when the electrical current was higher.

#### 3.3.2. Effects of the Amorphous Carbon Layer by Electron-Beam-Induced Deposition

Amorphous carbon deposition is inevitably generated during the nanostructure welding process, and the thickness of the amorphous carbon layer is positively correlated with the time of the e-beam irradiation, while negatively correlated with the degree of vacuum. As reported in the literature [21], when the top of the tungsten needle is only covered by a thin layer of amorphous carbon, the contact performance can be improved by the e-beam irradiation. Therefore, does the amorphous carbon layer possess other effects besides improving contact performance?

**Figure 9:**
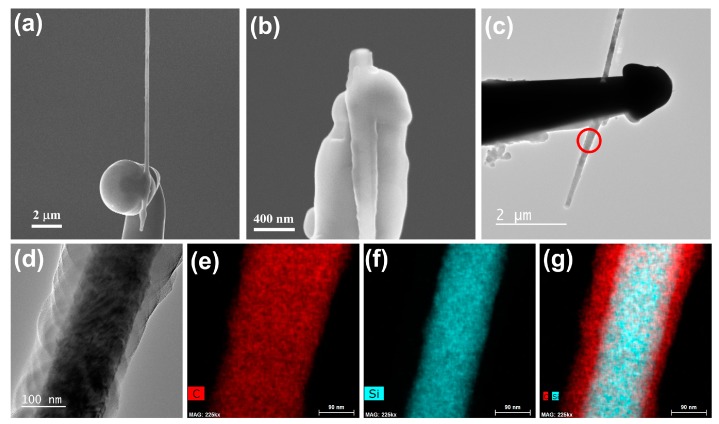
(**a**) SEM image of the individual SiC nanowire covered by an amorphous carbon layer at the welding area; (**b**) SEM image of the broken individual SiC nanowire welded onto the tungsten needle; (**c**) TEM image of the welded structure with low magnification; and (**d**) high-magnification TEM image of the area near the welding point marked by the red circle in figure (**c**). (**e**–**g**) energy-dispersive X-ray spectroscopy (EDX) mapping images: (**e**) carbon element distribution in the SiC nanowire; (**f**) silicon element distribution in the SiC nanowire; and (**f**) mixed silicon and carbon element distributions in the SiC nanowire.

In our experiment, we found that the amorphous carbon layer could also improve the mechanical performance of the welded joints. As shown in Figure 9, when the nanostructure welding process continued for more than 3 h, the thickness of the amorphous carbon layer was sufficient to cover the nanowire welding areas and, subsequently, a good mechanical contact was formed between the nanowire and tungsten needle. As described in our recent study [46], after fixing onto two tungsten needles by the nanostructure welding process, the piezoresistive effect of the SiC nanowire was characterized by in-situ tensile measurements. Furthermore, the results of the in-situ tensile measurements showed that the morphology of the welded joints remained in its original state even though the SiC nanowire was completely broken, as shown in Figure 9b. It can be seen that the mechanical performances of the welded joints were superior to that of the SiC nanowire. On the contrary, when the surface of the welded joint was not covered by a sufficiently thick amorphous carbon layer, the SiC nanowire could be easily removed from the top of the tungsten needle. To confirm that the material covered on the welded joints was an amorphous carbon layer, we performed energy-dispersive X-ray spectroscopy (EDX) mapping on the cover layer. However, due to the limited penetration depth of the TEM, it was impossible to directly analyze the structure and composition of the welded joints. Thus, we selected the parts of the nanowire near the welded joint for analysis, which is marked by a red circle in Figure 9c. The results of the EDX analysis are shown in Figure 9e–g. As can be seen from Figure 9d, the SiC nanowire was wrapped by an amorphous layer and the EDX analysis results indicate that the amorphous layer was mainly composed of carbon. The above results demonstrate that the amorphous carbon layer deposited by the e-beam irradiation benefitted the mechanical performance of the welded joints.

## 4. Conclusions

In summary, a universal method to realize nanostructure welding between individual 1D nanostructures and tungsten needles was presented in this paper. By adjusting the welding process, we can obtain individual welded 1D nanostructures with different orientations. Furthermore, the results of the in-situ electrical measurements done with SEM show that five different types of individual 1D nanostructures (LaB_6_ nanowire, CNT, ZnO nanoneedle, SiC nanowire, and CuO nanowire) established good Ohmic contacts with the tungsten needles. To understand the effects of e-beam irradiation and an electrical current flowing through the welded joint during the nanostructure welding process, the finite element method was used to simulate the local heating effects and temperature distributions of the welded joints. The simulated results show that the temperature rise induced by the local heating effect from the electrical current is much higher than that induced by the heating effect of the e-beam irradiation. In addition, the temperature of the welded joint is also positively correlated with the electrical current. When the resistance of a semiconductive nanowire was too large to limit the electrical current, the welded joint was converted into a Schottky contact. Finally, the in-situ tensile measurements indicate that the welded joints obtained by the nanostructure welding method can achieve better mechanical performance than the nanowire itself exhibits.
